# Efficacy and safety of different insulin infusion methods in the treatment of total parenteral nutrition-associated hyperglycemia: a systematic review and network meta-analysis

**DOI:** 10.3389/fnut.2023.1181359

**Published:** 2023-08-03

**Authors:** Lu Cao, Dan Zhang, Ying Zhao, Nan Zhou, Peng Zhang

**Affiliations:** Department of Pharmacy, Shaanxi Provincial People’s Hospital, Xi’an, China

**Keywords:** network meta-analysis, insulin infusion method, hyperglycemia, TPN, hypoglycemia

## Abstract

**Aims:**

To systematically evaluate the efficacy and safety of different insulin infusion methods in the treatment of total parenteral nutrition (TPN)-associated hyperglycemia based on published literature and the data of completed clinical trials using a network meta-analysis.

**Methods:**

A comprehensive search of PubMed, Elsevier, Web of Science, EMBASE, Medline, clinicaltrials.gov, Cochrane Library, and three Chinese databases (Wanfang Data, China National Knowledge Infrastructure, and SINOMED) up to December 15, 2022, was performed to collect information on different insulin infusion methods used for the treatment of TPN-associated hyperglycemia, and the Cochrane systematic review method was used to screen the literature, evaluate the quality of the included literature, and extract clinical characteristics for a network meta-analysis. Clinical outcomes included mean blood glucose (MBG), hypoglycemia, hospital length of stay, hyperglycemia, surgical site infection (SSI) and mean total daily insulin.

**Results:**

A total of 21 articles, including 1,459 patients, were included to analyze 6 different routes of insulin infusion, including continuous intravenous insulin infusion (CVII), continuous subcutaneous insulin infusion (CSII), subcutaneous glargine insulin (s.c. GI), the addition of regular insulin to the PN mixture (RI-in-PN), multiple subcutaneous insulin injections (MSII) and 50% of insulin administered as RI-in-PN + 50% of insulin administered as s.c. GI (50% RI-in-PN + 50% s.c. GI). The results of the network meta-analysis showed that MSII was the least effective in terms of MBG, followed by CVII. The 6 interventions were basically equivalent in terms of the hypoglycemia incidence. In terms of the length of hospital stay, patients in the CVII group had the shortest hospital stay, while the MSII group had the longest. CVII was the best intervention in reducing the incidence of hyperglycemia. The incidence of SSI was the lowest in the CSII and CVII groups, and the mean daily insulin dosage was the lowest in the CVII group.

**Conclusion:**

Current literature shows that for the treatment of TPN-associated hyperglycemia, CVII is the most effective, reducing the incidence of hyperglycemia and shortening the length of hospital stay without increasing the incidence of hypoglycemia. MSII has the worst efficacy, leading to a higher MBG and longer hospital stay, and RI-in-PN, CSII, s.c. GI and 50% RI-in-PN + 50% s.c. GI are better in terms of efficacy and safety and can be substituted for each other.

**Systematic Review Registration:**

https://www.crd.york.ac.uk/prospero/, identifier CRD42023439290.

## 1. Introduction

Hyperglycemia is a very common healthcare problem in hospitalized patients. Studies have shown that up to 30% of inpatients receive some type of nutrition support and that the incidence of hyperglycemia is higher in these patients (1). Among them, patients receiving total parenteral nutrition (TPN), regardless of a previous history of diabetes (2), have increased insulin resistance due to elevated levels of corticosteroids and catecholamines, which are caused by elevated levels of organismal stress, acute illness or surgery (3, 4); increased supply of carbohydrates (5); the use of exogenous corticosteroids/vasopressor drugs (6, 7); and the loss of insulin-release regulation by incretin hormones (8). The incidence of hyperglycemia in patients receiving TPN can reach 44%–90% (7, 9, 10).

Several previous studies had shown that among inpatients receiving parenteral nutrition (PN), the risk of infection, cardiac complications, acute renal failure, respiratory failure and even death increased as mean blood glucose (MBG) rose (4, 7, 11, 12), and in patients treated with PN, there is a significant linear relationship between the incidence of adverse outcomes and MBG once the blood glucose exceeded 113 mg/dL (13), with a 1.58-fold increase in the risk of any complication for every 18 mg/dL increase in blood glucose above this threshold (14). A prospective multicenter study conducted by Olveria et al. (11) enrolling 605 patients showed that compared to patients with MBG <140 mg/dL, the mortality rates among patients receiving PN who had blood glucose levels above 180 mg/dL increased 5.6-fold. However, excessive glycemic control itself increases the risk of hypoglycemia, which is likewise associated with poor clinical outcomes (7). Therefore, managing blood glucose in patients receiving PN is important for the prognosis of this population. The American Society for Parenteral and Enteral Nutrition (ASPEN) clinical guidelines and the American Diabetes Association (ADA) recommend a glycemic control target of 140–180 mg/dL for hospitalized adult patients receiving PN (2, 15).

The guidelines recommend the use of insulin to control PN-induced hyperglycemia (16), while there are various routes of insulin administration, including continuous intravenous insulin infusion (CVII), continuous subcutaneous insulin infusion (CSII), subcutaneous long-acting insulin infusion, such as glargine insulin (GI), multiple subcutaneous insulin injections (MSII), the addition of regular insulin to the PN mixture (RI-in-PN), or any combination of these methods (4, 9, 17–20), and there are different advantages and disadvantages (18, 21, 22). Unfortunately, clinical guidelines do not explicitly provide recommendations on the optimal route and type of insulin therapy for PN-related hyperglycemia (6, 18, 23), and there is little literature comparing the efficacy and safety of these regimens. Thus, glycemic management during PN therapy remains very challenging in daily clinical practice for inpatients with or without diabetes.

This study was designed to evaluate the effect of different insulin infusion routes on clinical outcomes in the treatment of TPN-related hyperglycemia by means of a network meta-analysis to clarify the clinical efficacy and safety between different insulin infusion routes, with the aim of providing evidence-based medical evidence for the glycemic management of adult inpatients receiving TPN.

## 2. Materials and methods

We conducted a systematic review and network meta-analysis according to the preferred reporting items for systematic reviews and meta-analyses (PRISMA) extension statement for network meta-analysis (NMA) published in 2015 (see Appendix for PRISMA-NMA checklist).

### 2.1. Inclusion criteria

#### 2.1.1. Population

Adult inpatients aged ≥18 years who fasted and received TPN treatment for any reason.

#### 2.1.2. Interventions and comparisons

Insulin alone was used to control blood glucose, and different routes of insulin infusion included RI-in-PN, CVII, MSII, CSII, subcutaneous long-acting insulin, or any combination of the above regimens. The experimental group used one of the above regimens, and the control group used the other of the above regimens. The initial insulin dosage was based on the patient’s condition, previous insulin use (with diabetes), TPN formula, and the patient’s initial blood glucose, and the corrected insulin dosage was based on the patient’s blood glucose changes during TPN treatment, regardless of the disease type.

#### 2.1.3. Clinical outcomes

MBG; hypoglycemia; hospital length of stay; hyperglycemia; surgical site infection (SSI); mean total daily insulin. Hypoglycemia was defined as blood glucose levels <70 mg/dL (3, 4, 22). Hyperglycemia was defined as blood glucose levels >180 mg/dL (3, 9).

#### 2.1.4. Study design

Published randomized controlled trials (RCTs) and prospective or retrospective studies.

### 2.2. Exclusion criteria

(i) Concurrent enteral nutrition (EN) or oral feeding; (ii) concurrent autoimmune diseases, impaired liver function (serum aspartate aminotransferase or alanine aminotransferase levels more than 3 times normal) (9) or impaired renal function (glomerular filtration rate less than 45 mL/min) (22); (iii) pregnancy; (iv) diagnosis of type I diabetes; (v) concurrent use of corticosteroids or vasopressors; (vi) insufficient clinical outcome data, or no accurate definition of hypoglycemia and hyperglycemia, or a definition of hypoglycemia and hyperglycemia inconsistent with our definition; (vii) duplicated publications; (viii) case reports; (ix) conference abstracts; (x) inability to download the full text or inability to obtain the full text after contacting the author; (xi) non-Chinese and English literature.

### 2.3. Search strategy

All studies were identified by a systematic review of databases, including PubMed, Elsevier, Web of Science, EMBASE, Medline, clinicaltrials.gov, the Cochrane Library, and three Chinese databases (Wanfang Data, China National Knowledge Infrastructure, and SINOMED) up to December 15, 2022 using the following terms: (“parenteral nutrition” OR “nutrition, parenteral” OR “parenteral feeding*” OR “feeding*, parenteral” OR “intravenous feeding*” OR “feeding*, intravenous”) AND (“insulin” OR “insulin, regular” OR “regular insulin” OR “soluble insulin” OR “insulin, soluble” OR “insulin A chain” OR “sodium insulin” OR “insulin, sodium” OR “novolin” OR “iletin” OR “insulin B chain” OR “chain, insulin B”). A search method combining subject headings and free text was used, and adjustments were made according to the specific databases. The reference lists of the included papers and previous reviews were manually screened to identify additional studies.

### 2.4. Literature selection and quality assessment

Two investigators (LC and NZ) independently screened the publications, performed quality assement on the preliminary included literature which were in line with the PICOS (population, intervention, comparison, outcome and study design) criteria, and cross-checked the results. Any disagreements were resolved by consensus or consultation with a third investigator. RCTs were assessed using the adjusted Jadad scale (24). Assessment parameters included the randomization method, allocation concealment, blinding, and patient loss to follow-up or withdrawal. The total score was 7, and studies with a score between 1 and 3 were considered low-quality studies, while those with a score between 4 and 7 were considered high-quality studies. Retrospective studies were evaluated with the Newcastle–Ottawa scale (NOS) (25), which assesses the representativeness of participants, comparability of participants, follow-up and assessment of follow-up sufficiency, and patient loss to follow-up or withdrawal. Studies with a score between 5 and 9 were considered to have less bias and were of high quality. Finally, the high-quality studies were included in the network meta-analysis.

### 2.5. Data extraction of the included studies

Two investigators (LC and NZ) independently extracted the data, and cross-checked the results. Any disagreements were resolved by consensus or consultation with a third investigator. The following data were extracted independently: (i) basic information of the publications (first author, publication year, and type of study); (ii) clinical characteristics of the patients (age, number of patients, disease type, and whether critical illness); (iii) interventions; (iv) TPN (course of treatment, total energy, macronutrient content, etc.); (v) clinical outcomes (MBG; hypoglycemia; hospital length of stay; hyperglycemia; SSI; mean total daily insulin); and (vi) quality assessment indicators.

### 2.6. Statistical analysis

Network meta-analysis under the frequency framework was implemented using the network and mvmeta commands of Stata 15.1 software fitting a multivariate random-effects meta-analysis model using the restricted maximum likelihood. The network diagram was drawn as a simple summary description to show all available evidence for the various interventions. In the studies, if the clinical outcomes were continuous variables, the weighted mean difference (WMD) and its 95% confidence interval (CI) were utilized; for dichotomous variables, the relative risk (RR) and its 95% CI were employed. Initially, we explored the transitivity assumption by comparing the distribution of potential effect modifiers across studies (initial blood glucose, age, gender, etc.) and performed random effects frequentist network meta-analysis. Subsequently, the global heterogeneity was evaluated with generalised methods of moments estimate of variance between studies andtested by the design based decomposition of Cochran’s *Q* statistic. Consistency and inconsistency model tests were performed on the data to determine the inconsistency of the overall network, and *p* < 0.05 was considered to indicate the presence of inconsistency. The node splitting method was used to check the local inconsistency of each node of the network diagram, and local inconsistency was considered if *p* < 0.05. Inconsistency tests were performed when there was a closed loop, and the inconsistency factors (IF), 95% CI and the *p*-value of its *Z* test were calculated. When *p* > 0.05, the lower limit of the 95% CI of the IF value equal to 0 was considered good consistency between the direct and indirect comparison results; otherwise the closed loop was considered to have significant inconsistency. The results of pairwise comparisons between different interventions are presented in league tables. Each intervention was ranked using the surface under the cumulative ranking curve (SUCRA), with higher SUCRA values indicating a greater likelihood that a treatment regimen was at the highest level or highly effective, resulting in the best intervention for outcome measures. Publication bias and small-study effects tests for the clinical outcomes were completed to generate comparison-adjusted funnel plots, and whether the distribution of scatter was symmetrical was qualitatively judged to detect whether the literature included in the network meta-analysis had publication bias.

## 3. Results

### 3.1. Literature search results and quality assessment of included studies

**Figure 1:**
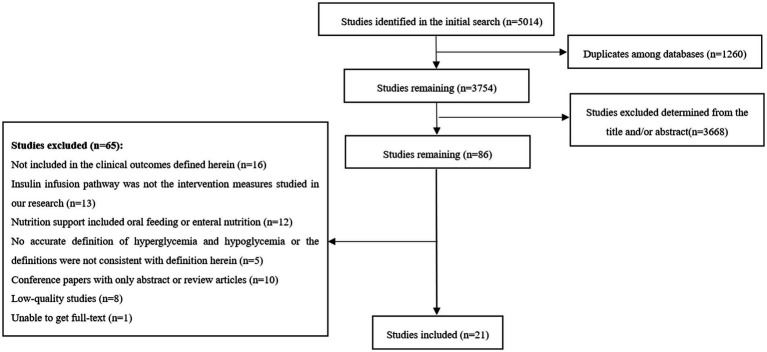
Flow diagram of the study selection process.

The proposed search terms were searched in the database, and a total of 5,014 documents were identified, including 3,828 in English and 1,186 in Chinese. Finally, 21 studies were included in the network meta-analysis. The literature screening process was presented in Figure 1. Of the 21 included papers, 17 studies (3, 9, 22, 26–39) were RCTs, and the methodological evaluation of the adjusted Jadad scale showed that they were of high quality (4–6 points); the remaining 4 articles (4, 40–42) were retrospective studies with NOS scores of 6–9 points. The overall methodological quality was fair, and the specific scores were shown in Table 1.

**Table 1:** Characteristics and quality assessments of the included studies.

Author, year	Age /years	Study design	Population	Critical illness	Blood glucose control targets/mg/dL	Durations of TPN/days	Interventions		Clinical outcomes	Adjusted Jadad /NOS scale
							Experimental group	Control group		
Wu, 2022 (42)	≥18	A retrospective single center study	Patients undergoing PD surgery	NO	144–180	≥5	RI-in-PN (*n* = 52)	CVII (*n* = 63)	③ ④ ⑤	8
Olveira, 2020 (22)	≥18	A prospective multicenter randomized open-label study	Hospitalized noncritically ill type 2 diabetes patients	NO	70–180	≥5	RI-in-PN (*n* = 80)	50% RI-in-PN + 50% s.c. GI (*n* = 81)	① ② ③ ⑤ ⑥	4
Truong, 2019 (4)	≥18	A retrospective chart review	Patients with PN-induced hyperglycemia after general surgery	NO	<180	≥3	RI-in-PN (*n* = 78)	s.c. GI (*n* = 35)	② ③	9
Li, 2018 (3)	18–80	A single-center, prospective, randomized, open-label trial	Patients with type 2 diabetes undergoing gastrointestinal surgery	NO	70–180	≥4	CSII (*n* = 50)	MSII (*n* = 52)	①	5
Wang, 2018 (39)	≥18	A single-center, prospective, RCT study	Perioperatively fasting patients with diabetes	NO	72–250	NA	CSII (*n* = 25)	s.c. GI (*n* = 22)	① ④	4
Hakeam, 2017 (9)	≥18	A single-center, prospective randomized open-label study	Diabetic patients undergoing noncardiac surgery	NO	140–234	≥7	RI-in-PN (*n* = 32)	s.c. GI (*n* = 35)	② ④	4
Yang, 2016 (41)	≥18	A retrospective single center study	Diabetic patients undergoing abdominal surgery	NO	144–180	≥4	RI-in-PN (*n* = 12)	CVII (*n* = 15)	②	8
He, 2016 (38)	58.46 ± 4.2	A single-center, prospective, RCT study	Patients with PN-induced hyperglycemia after PD surgery	NO	Fasting: 108–144 Food intake: 79–180	NA	RI-in-PN (*n* = 70)	CVII (*n* = 106)	③ ⑤	5
Oghazian, 2015 (37)	≥18	A prospective, randomized, open-label, controlled trial	Postoperative patients admitted to the ICU	YES	110–180	≥7	RI-in-PN (*n* = 21)	s.c. GI (*n* = 21)	② ③ ④	4
Shi, 2015 (36)	45–67	A single-center, prospective, RCT study	Diabetic patients undergoing gastric cancer surgery	NO	NA	≥3	RI-in-PN (*n* = 25)	CVII (*n* = 25)	③	5
Neff, 2014 (40)	≥18	A retrospective single center study	Patients with PN-induced hyperglycemia	NO	72–180	NA	CVII (*n* = 32)	MSII (*n* = 21)	① ② ③	6
Duan, 2013 (35)	29–93	A single-center, prospective, RCT study	Diabetic patients undergoing abdominal surgery	NO	108–216	≥7	RI-in-PN (*n* = 30)	CVII (*n* = 30)	② ⑥	5
Liu, 2012 (34)	57.4	A single-center, prospective, RCT study	Diabetic patients undergoing gastric cancer surgery	NO	108–198	NA	RI-in-PN (*n* = 24)	CSII (*n* = 21)	① ③	5
Gao, 2011 (32)	54.1	A single-center, prospective, RCT study	Diabetic patients undergoing gastric cancer surgery	NO	NA	≥3	RI-in-PN (*n* = 21)	CVII (*n* = 24)	③	5
Zheng, 2011 (33)	48–79	A single-center, prospective, RCT study	Fasting patients with diabetes	NO	NA	NA	CSII (*n* = 29)	s.c. GI (*n* = 29)	① ②	4
Lan, 2010 (30)	35–85	A single-center, prospective, RCT study	Diabetic patients undergoing gastrointestinal tumor surgery	NO	108–198	NA	RI-in-PN (*n* = 48)	CVII (*n* = 43)	③ ⑤ ⑥	5
Wang, 2010 (31)	50.1	A single-center, prospective, RCT study	Diabetic patients undergoing gastric cancer surgery	NO	108–180	≥3	RI-in-PN (*n* = 21)	CVII (*n* = 24)	③	5
Huang, 2009 (28)	51.8 ± 11.5	A single-center, prospective, RCT study	Fasting patients with diabetes	NO	NA	NA	CSII (*n* = 28)	MSII (*n* = 28)	① ③	4
Long, 2009 (29)	≥18	A single-center, prospective, RCT study	Nondiabetic patients undergoing upper gastrointestinal tumor surgery	NO	72–216	≥3	RI-in-PN (*n* = 10)	CVII (*n* = 10)	②	6
Chen, 2007 (27)	21–70	A single-center, prospective, RCT study	Fasting patients with diabetes	NO	NA	NA	CSII (*n* = 23)	MSII (*n* = 23)	① ② ③	4
Han, 2006 (26)	≥18	A single-center, prospective, RCT study	Diabetic patients undergoing abdominal surgery	YES	100–150	≥7	CSII (*n* = 20)	CVII (*n* = 20)	② ⑤	5

PD, pancreaticoduodenectomy; NA, not available. Clinical outcomes: ① mean blood glucose; ② hypoglycemia; ③ hospital length of stay; ④ hyperglycemia; ⑤ surgical site infection; ⑥ mean total daily insulin.

### 3.2. Basic characteristics of the included studies

Twenty-one studies (3, 4, 9, 22, 26–42) included 1,459 patients, consisting of 1,377 noncritically ill patients and 82 critically ill patients, and they included 6 different interventions, of which 524 were RI-in-PN, 392 were CVII, 124 were MSII, 196 were CSII, 142 were s.c. GI, and 81 were 50% RI-in-PN + 50% insulin glargin. Each study designed the experimental and control groups with consideration of the basic patient profiles and disease types, and the two groups were comparable at baseline. The total energy and macronutrient content of the TPN were not reported in the vast majority of studies. Most of the study subjects were perioperative patients receiving the following types of surgery: gastrointestinal surgery, pancreaticoduodenectomy, cardiac surgery and so on. The basic characteristics of the included studies were shown in Table 1.

### 3.3. Network diagram

**Figure 2:**
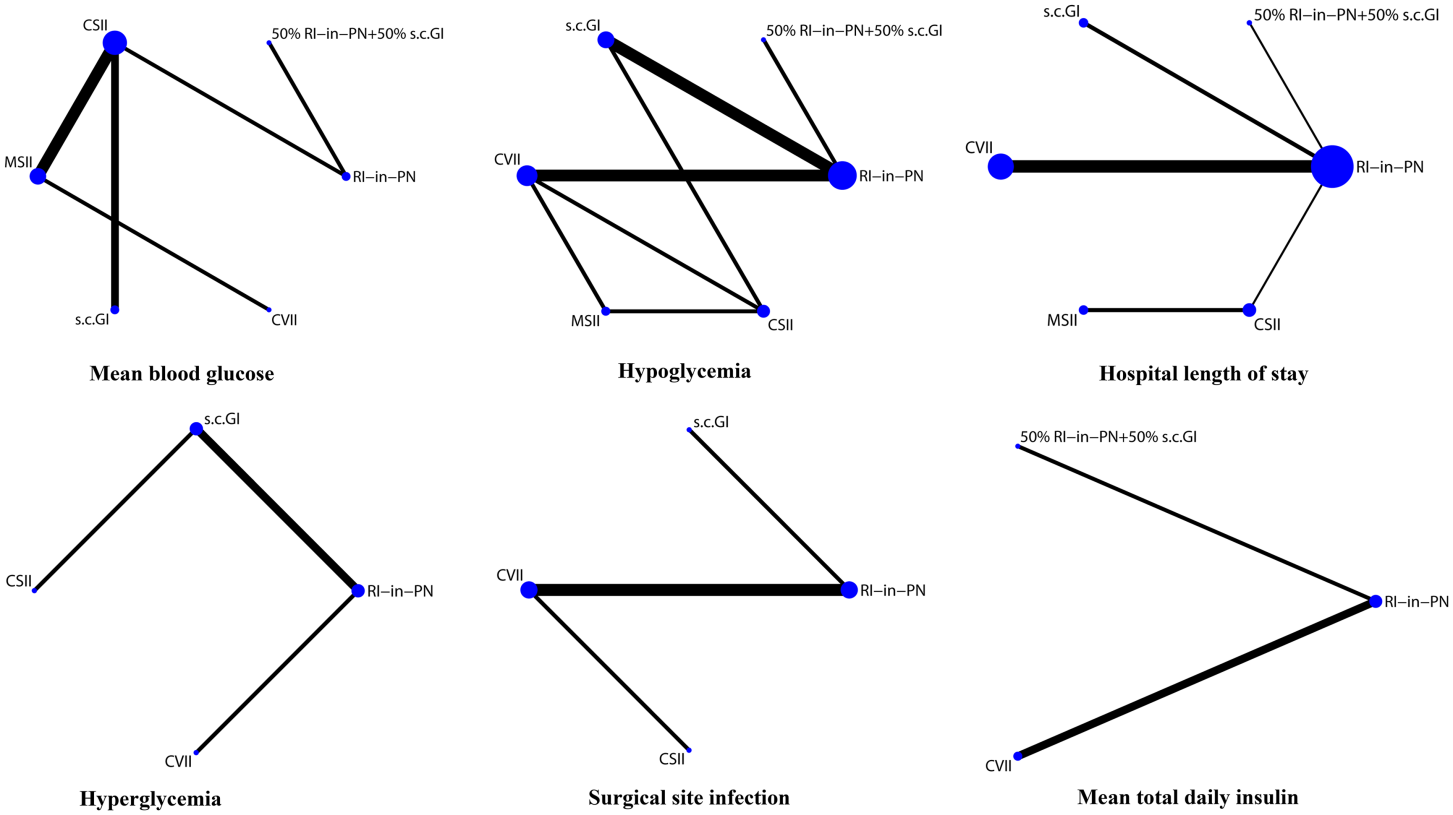
Evidence structure of interventions.

Visual network geometry diagrams were used to show each intervention and the correlation between them. Nodes indicated the interventions and number of patients, and the more patients included in the study, the larger were the nodes. For interventions with solid line links compared with each other, the more studies included, the thicker were the solid lines. A total of 21 papers containing 6 interventions were included in this study, and a network diagram of clinical outcomes was drawn according to the preset outcome indicators, as shown in Figure 2.

### 3.4. Consistency test

According to the network diagram, no closed loop was formed in the comparison of MBG, hospital length of stay, hyperglycemia, SSI and mean total daily insulin, and a global inconsistency test was not needed. The results of the nodal splitting method of the local inconsistency test showed that all *p*-values were >0.05, indicating that there was no significant local inconsistency in our study. The comparison results were all from direct comparisons, and an inconsistency test was not required to assess the differences between direct comparison and indirect comparison results. The incidence of hypoglycemia formed 2 closed loops: (1) RI-in-PN-s.c. GI-CVII-CSII; (2) CVII-MSII-CSII, with a global inconsistency test result of *p* > 0.05, indicating the presence of consistency, and the local inconsistency test result of *p*-value >0.05 for both closed loops indicated that there was no significant local inconsistency in the study. The inconsistency test results showed IF = 3.982 (95% CI: 0.00–7.36, *p* = 0.521) for the first closed loop and IF = 1.232 (95% CI, 0.00–4.39, *p* = 0.444) for the second closed loop, indicating that the results of direct and indirect comparisons of these 2 closed loops were in good agreement.

### 3.5. Results of network meta-analysis

#### 3.5.1. Mean blood glucose

A total of 8 studies (3, 22, 27, 28, 33, 34, 39, 40) reported differences in MBG, including 6 interventions, and the results of their pairwise comparisons were shown in Table 2. The league table showed that the MBG in the MSII group was significantly higher than that in the s.c. GI, CSII, RI-in-PN, 50% RI-in-PN + 50% s.c. GI and CVII groups. MBG in the CVII group was also significantly higher than that in the s.c. GI and CSII groups. There were no statistically significant differences between the remaining interventions. That was, MSII had the poorest control of MBG, followed by CVII, while the four interventions of s.c. GI, CSII, RI-in-PN, and 50% RI-in-PN + 50% s.c. GI were generally comparable in controlling MBG.

**Table 2:** League table of mean blood glucose of different interventions.

**s.c. GI**	0.11 (−0.48, 0.70)	0.51 (−0.77, 1.79)	0.91 (−0.54, 2.36)	**1.64 (0.12, 3.17)**	**3.24 (2.50, 3.99)**
−0.11 (−0.70, 0.48)	**CSII**	0.40 (−0.73, 1.53)	0.80 (−0.52, 2.12)	**1.53 (0.13, 2.94)**	**3.13 (2.68, 3.59)**
−0.51 (−1.79, 0.77)	−0.40 (−1.53, 0.73)	**RI-in-PN**	0.40 (−0.28, 1.08)	1.13 (−0.67, 2.94)	**2.73 (1.51, 3.95)**
−0.91 (−2.36, 0.54)	−0.80 (−2.12, 0.52)	−0.40 (−1.08, 0.28)	**50% RI-in-PN+50% s.c. GI**	0.73 (−1.19, 2.66)	**2.33 (0.94, 3.73)**
**−1.64 (−3.17, −0.12)**	**−1.53 (−2.94, −0.13)**	−1.13 (−2.94, 0.67)	−0.73 (−2.66, 1.19)	**CVII**	**1.60 (0.27, 2.93)**
**−3.24 (−3.99, −2.50)**	**−3.13 (−3.59, −2.68)**	**−2.73 (−3.95, −1.51)**	**−2.33 (−3.73, −0.94)**	**−1.60 (−2.93, −0.27)**	**MSII**

#### 3.5.2. Hypoglycemia

Eleven studies (4, 9, 22, 26, 27, 29, 33, 35, 37, 40, 41) reported differences in the incidence of hypoglycemia, including 6 interventions, and the results of their two-by-two comparisons were shown in Table 3. The league table showed no statistically significant differences between the 6 interventions in the incidence of hypoglycemia. That was, the incidence of hypoglycemia caused by these 6 interventions of MSII, CVII, s.c. GI, CSII, RI-in-PN, and 50% RI-in-PN + 50% s.c. GI in the treatment of PN-associated hyperglycemia was generally comparable.

**Table 3:** League table of hypoglycemia of different interventions.

**CSII**	1.91 (0.36, 10.03)	2.07 (0.37, 11.62)	2.29 (0.41, 12.72)	2.36 (0.39, 14.26)	5.70 (0.46, 71.34)
0.52 (0.10, 2.76)	**MSII**	1.09 (0.23, 5.10)	1.20 (0.16, 8.82)	1.24 (0.18, 8.48)	2.99 (0.22, 40.96)
0.48 (0.09, 2.71)	0.92 (0.20, 4.33)	**CVII**	1.11 (0.20, 6.06)	1.14 (0.26, 4.91)	2.75 (0.28, 27.42)
0.44 (0.08, 2.42)	0.83 (0.11, 6.10)	0.90 (0.16, 4.94)	**s.c. GI**	1.03 (0.30, 3.50)	2.49 (0.29, 21.45)
0.42 (0.07, 2.56)	0.81 (0.12, 5.53)	0.88 (0.20, 3.78)	0.97 (0.29, 3.30)	**RI-in-PN**	2.41 (0.41, 14.25)
0.18 (0.01, 2.20)	0.33 (0.02, 4.59)	0.36 (0.04, 3.62)	0.40 (0.05, 3.47)	0.41 (0.07, 2.44)	**50% RI-in-PN+50% s.c. GI**

#### 3.5.3. Hospital length of stay

A total of 13 studies (4, 22, 27, 28, 30–32, 34, 36–38, 40, 42) reported differences in hospital length of stay; however, the inconsistency test resulted in *p* < 0.05, and examination of the data revealed that the data from Neff et al. (40) were highly heterogeneous and did not conform to a normal distribution compared with data from other studies and were therefore excluded. The remaining 12 studies (4, 22, 27, 28, 30–32, 34, 36–38, 42) were included to continue the network meta-analysis, containing a total of 6 interventions, and the results of their pairwise comparisons were shown in Table 4. The league table showed that the CVII group had a shorter hospital length of stay than the RI-in-PN, s.c. GI, CSII and MSII groups, with statistically significant differences. The MSII group had a significantly longer hospital length of stay than the RI-in-PN, s.c. GI and CSII groups. The differences between the remaining interventions were not statistically significant. Therefore, CVII was the most effective and MSII the least effective in reducing hospital length of stay, and it was generally comparable between the four interventions of s.c. GI, CSII, RI-in-PN, and 50% RI-in-PN + 50% s.c. GI.

**Table 4:** League table of hospital length of stay of different interventions.

**CVII**	1.44 (−6.30, 9.17)	**2.74 (1.81, 3.66)**	**2.83 (0.46, 5.20)**	**3.34 (0.98, 5.70)**	**9.14 (5.99, 12.29)**
−1.44 (−9.17, 6.30)	**50% RI-in-PN+50% s.c. GI**	1.30 (−6.38, 8.98)	1.40 (−6.59, 9.38)	1.90 (−6.08, 9.88)	7.70 (−0.55, 15.95)
**−2.74 (−3.66, −1.81)**	−1.30 (−8.98, 6.38)	**RI-in-PN**	0.10 (−2.09, 2.28)	0.60 (−1.57, 2.77)	**6.40 (3.39, 9.41)**
**−2.83 (−5.20, −0.46)**	−1.40 (−9.38, 6.59)	−0.10 (−2.28, 2.09)	**s.c. GI**	0.50 (−2.58, 3.58)	**6.30 (2.59, 10.02)**
**−3.34 (−5.70, −0.98)**	−1.90 (−9.88, 6.08)	−0.60 (−2.77, 1.57)	−0.50 (−3.58, 2.58)	**CSII**	**5.80 (3.72, 7.88)**
**−9.14 (−12.29, −5.99)**	−7.70 (−15.95, 0.55)	**−6.40 (−9.41, −3.39)**	**−6.30 (−10.02, −2.59)**	**−5.80 (−7.88, −3.72)**	**MSII**

#### 3.5.4. Hyperglycemia

Four studies (9, 37, 39, 42) reported differences in the incidence of hyperglycemia, including 4 interventions, with the results of their pairwise comparisons shown in Table 5. The league table showed that the incidence of hyperglycemia in the CVII group was significantly lower than that in the RI-in-PN, s.c. GI and CSII groups. There were no significant differences between the remaining interventions. Thus, CVII was the best at reducing the incidence of hyperglycemia, and the three interventions of s.c. GI, RI-in-PN, and CSII were similar.

**Table 5:** League table of hyperglycemia of different interventions.

**CVII**	**1.79 (1.59, 2.02)**	**1.82 (1.19, 2.79)**	**1.86 (1.17, 2.95)**
**0.56 (0.50, 0.63)**	**RI-in-PN**	1.01 (0.67, 1.53)	1.04 (0.66, 1.62)
**0.55 (0.36, 0.84)**	0.99 (0.65, 1.49)	**s.c. GI**	1.02 (0.86, 1.21)
**0.54 (0.34, 0.85)**	0.96 (0.62, 1.51)	0.98 (0.82, 1.16)	**CSII**

#### 3.5.5. Surgical site infection

A total of 5 studies (22, 26, 30, 38, 42) reported differences in the incidence of SSI, including 4 interventions, and the results of their two-by-two comparisons were shown in Table 6. The league table showed that the CSII group had a significantly lower incidence of SSI than the s.c. GI and RI-in-PN groups. The CVII group had a significantly lower incidence than the RI-in-PN group. There were no significant differences between the other interventions. CSII and CVII were more effective in reducing the incidence of SSI, followed by s.c. GI and RI-in-PN.

**Table 6:** League table of surgical site infection of different interventions.

**CSII**	7.00 (0.95, 51.80)	**12.85 (1.11, 148.53)**	**20.82 (2.31, 187.75)**
0.14 (0.02, 1.06)	**CVII**	1.84 (0.45, 7.51)	**2.97 (1.20, 7.40)**
**0.08 (0.01, 0.90)**	0.54 (0.13, 2.23)	**s.c. GI**	1.62 (0.55, 4.74)
**0.05 (0.01, 0.43)**	**0.34 (0.14, 0.84)**	0.62 (0.21, 1.81)	**RI-in-PN**

#### 3.5.6. Mean total daily insulin

A total of 3 studies (22, 30, 35) reported differences in mean total daily insulin, including 3 interventions, and the results of their pairwise comparisons were shown in Table 7. The league table showed that the CVII group had a significantly lower mean total daily insulin dosage than both the RI-in-PN and 50% RI-in-PN + 50% s.c. GI groups. The differences between the remaining interventions were not statistically significant. CVII was most effective in reducing the mean total daily insulin dosage.

**Table 7:** League table of mean total daily insulin of different interventions.

**CVII**	**13.89 (9.09, 18.68)**	**18.59 (7.44, 29.73)**
**−13.89 (−18.68, −9.09)**	**RI-in-PN**	4.70 (−5.36, 14.76)
**−18.59 (−29.73, −7.44)**	−4.70 (−14.76, 5.36)	**50% RI-in-PN+50% s.c. GI**

Among the six clinical outcomes, MBG, hospital length of stay and mean total daily insulin were continuous variables, and hypoglycemia, hyperglycemia and SSI were dichotomous variables. The league tables were the results of pairwise comparisons between different interventions where statistically significant pair-wise comparisons of interventions were highlighted in bold. Reading from left to right, both WMD and its 95% CI were <0 or both RR and its 95% CI were <1 indicating a statistically significant difference.

### 3.6. Ranking of intervention efficacy

**Figure 3:**
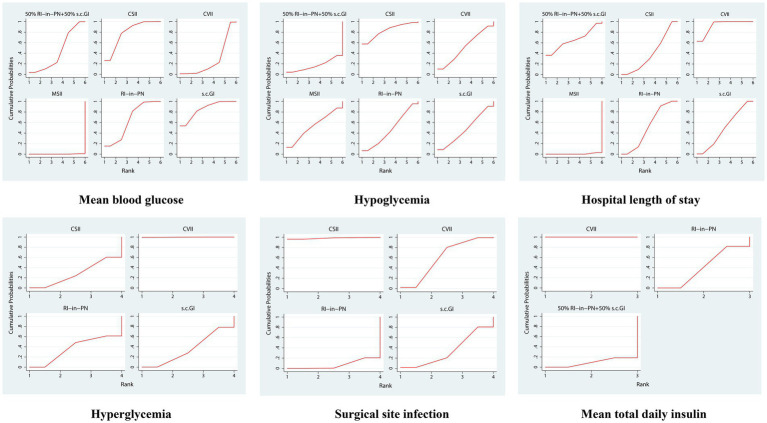
SUCRA in the change of different intervention measures.

To visualize the effects of the interventions on clinical outcomes, SUCRA curves were drawn to rank each intervention, as shown in Figure 3. The ranking for MBG, from lowest to highest, was as follows: s.c. GI (85.8%), CSII (79.5%), RI-in-PN (64.5%), 50% RI-in-PN + 50% s.c. GI (42.5%), CVII (27.4%), MSII (0.2%). In terms of reducing the incidence of hypoglycemia, the best intervention was CSII (83.3%), followed by MSII (53.4%), CVII (51.8%), s.c. GI (47.7%), RI-in-PN (47.0%), 50% RI-in PN + 50% s.c. GI (16.9%). The ranking for hospital length of stay, in order from shortest to longest, was as follows: CVII (92.5%), 50% RI-in-PN + 50% s.c. GI (65.8%), RI-in-PN (65.8%), s.c. GI (49.3%), CSII (39.8%), MSII (0.6%). In reducing the incidence of hyperglycemia, the best intervention was CVII (99.8%), followed by RI-in-PN (36.5%), s.c. GI (35.5%), CSII (28.2%). The best intervention in reducing the incidence of SSI was CSII (98.2%), followed by CVII (60.4%), s.c. GI (34.4%), and RI-in-PN (7.0%). The ranking for mean total daily insulin, from lowest to highest, was as follows: CVII (100.0%), RI-in-PN (40.8%), and 50% RI-in-PN + 50% s.c. GI (9.2%).

### 3.7. Publication bias

**Figure 4:**
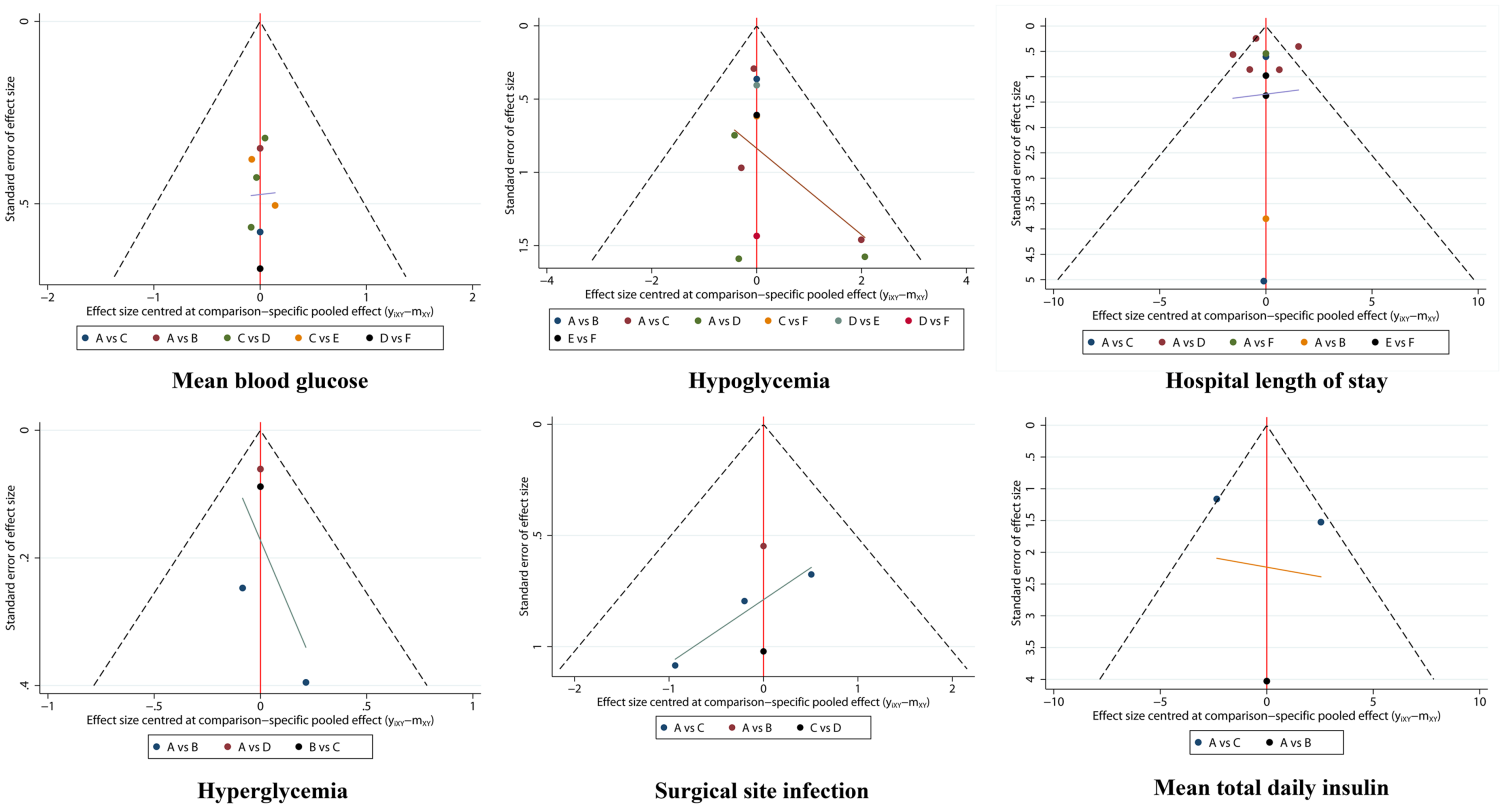
Comparison-adjusted funnel plots of interventions.

The comparison-adjusted funnel plot of each clinical outcome showed a relatively symmetrical distribution of the scattered points, indicating no significant publication bias or small-sample bias in this network meta-analysis, as shown in Figure 4.

## 4. Discussion

Hyperglycemia is very common in patients receiving PN support (1) and can increase the risk of complications such as infection, respiratory and renal complications, and even death (43), regardless of whether the patient has previous diabetes (2) or is admitted to the intensive care unit (ICU) (37). Treatment of PN-related hyperglycemia with insulin in turn predisposes patients to hypoglycemia (44, 45), which likewise predicts death and poorer clinical outcomes (37, 46). In actual clinical practice, due to the lack of monitoring equipment, insufficient nursing staff, and low frequency of blood glucose monitoring, undetectable hyperglycemic or hypoglycemic events often occur clinically, which significantly affects the prognosis of inpatients receiving PN; therefore, blood glucose management in these inpatients is critically necessary (16). Several observational studies have shown that glycemic management can be performed by changing the nutrient composition or by insulin infusion during TPN (17).

Currently, there are several insulin infusion methods to control PN-associated hyperglycemia. The most widely used route of administration in clinical practice is RI-in-PN, which has the advantages that the nutrient solution can be used with carbohydrates to intravenously deliver insulin at a stable rate, reduce the risk of hypoglycemia (18) and decrease the time required for care (10). However, it also has certain limitations, such as the need for strict asepsis. Moreover, the efficacy of insulin in PN may be reduced or unstable due to the materials composing the PN bag, and sugar-salt mixtures and acidity affect insulin activity and thus its concentration, causing fluctuations in patients’ blood glucose (47–49). CSII is a more common insulin infusion method in clinical practice that can simulate the physiological insulin secretion pattern, thereby providing a more flexible insulin infusion and making it easier to control blood glucose changes and maintain them at the desired level (50); furthermore, CSII does not require multiple injections, which is more easily accepted by patients. However, this mode is expensive and requires certain operational skills, so there are some difficulties in its widespread implementation in primary hospitals (51). Similarly, CVII is a commonly used route of administration for controlling PN-associated hyperglycemia; the insulin can be rapidly absorbed and can rapidly correct hyperglycemia, making CVII the preferred insulin infusion method for critically ill or hemodynamically unstable patients (52). A large body of literature has shown that the use of CVII can be dynamically adjusted according to blood glucose values to achieve smoother glycemic control and avoid excessive fluctuations (17). However, there is no clear regulatory specification for CVII, and clinical use is mostly an empirical practice (16). The use of CVII requires more nursing time for patient care, and there is a risk of hypoglycemia if the PN is stopped without stopping insulin infusion (53). MSII and subcutaneous single injections of long-acting insulins, such as glargine insulin, are the traditional ways of controlling blood glucose, and these protocols are simple to perform but have disadvantages. MSII has a slow onset of action, yields poor glycemic control (51), and poses an increased risk of hypoglycemia if the patient is exposed to long-acting insulin when discontinuing PN (53). In recent years, the frequency of clinical use has been lower than that of other methods. Recently, there have been several studies on s.c. GI for controlling PN-associated hyperglycemia, the treatment can achieve similar glycemic control and safety, at least in theory, and it can reduce glycemic variability (9, 37) while being inexpensive and easy to perform (51). However, because these agents are long-acting insulins, they may lead to an increased risk of hypoglycemia in patients if PN infusions are completed on the same day (10). A new insulin infusion modality that has not been used before now exists. A multicenter randomized clinical trial (INSUPAR trial) conducted by Olveria et al. in 2020 evaluated the efficacy and safety of 50% RI-in-PN + 50% s.c. GI for glycemic control during TPN in noncritically ill inpatients with diabetes mellitus; the results showed that it was comparable to RI-in-PN without significant differences, but with better glycemic control after interruption of TPN and, notably, a higher incidence of hypoglycemia (22).

There are no clear clinical guidelines regarding the best type of insulin and infusion method to manage glycemia in patients receiving PN, as well as not much evidence-based medical evidence to validate a given glucose management strategy. Laesser et al. (10) published a systematic review in 2019 qualitatively describing glycemic management measures in noncritically ill inpatients receiving PN/EN. Verçoza Viana et al. (6) included 2 studies for meta-analysis to assess the efficacy of RI-in-PN and s.c. GI in the treatment of patients with PN-associated hyperglycemia and showed no significant differences in MBG and incidence of hypoglycemia between the two interventions. Our study is the first to quantitatively assess the safety and efficacy of six common insulin infusion modalities during PN by means of a network meta-analysis to improve glycemic management in inpatients receiving PN therapy.

The results of this study showed that MSII was the least effective intervention in terms of MBG, and patients in this group had the highest MBG, which was consistent with previously published studies (3, 27, 28, 40). In patients using MSII, it is difficult to mimic the normal physiological insulin secretion pattern due to the high variability in insulin absorption rates and the limited duration of insulin action; in addition, MSII requires multiple subcutaneous injections to patients, which increases pain and is not easily accepted by patients, resulting in poor compliance and making patients’ blood glucose prone to fluctuations and difficult to control (27, 28, 51). Compared with CVII, CSII exerts better control of patients’ MBG, and a prospective study conducted by Han et al. (26) including 40 diabetic patients undergoing major abdominal surgery also showed the same result; that is, CSII was better than CVII in controlling blood glucose levels, and it is believed that CSII treatment can mimic the physiological insulin secretion pattern of normal humans, which is mainly characterized by more stable insulin absorption by the body and smoother glycemic control (54). Patients in the CVII group had a higher MBG than those in the s.c. GI group, which could be explained by the comparable MBG between the s.c. GI and CSII. Moreover, CVII is rapidly absorbed, rapidly corrects hyperglycemia, and correspondingly will also be more likely to lead to the rapid occurrence of hypoglycemia (52). According to the research of Yang et al. (41), CVII as an interventional measure often produces greater blood glucose fluctuations due to the different insulin pump input rates set by nurses. At present, we adjust insulin micropumps according to traditional experience to control patients’ blood glucose, which requires frequent blood glucose monitoring, greatly increasing the workload of nurses. Generally, there are fewer nursing staff members in non-ICU wards, albeit with heavier workloads, and blood glucose monitoring there is not as intensive as it is in ICU wards. In view of the enormous harm of hypoglycemia, medical staff will be more cautious when using CVII compared with subcutaneous insulin in patients, selecting more conservative pump rates and doses to maintain the blood glucose at a higher level within the normal range and avoid the occurrence of hypoglycemia as much as possible. Therefore, the MBG in the CVII group will be relatively higher. According to the ranking of intervention efficacy in our findings, although the difference was not statistically significant, s.c. GI caused a higher incidence of hypoglycemia than CVII, also verifying this reason. Finally, the 4 interventions, s.c. GI, CSII, RI-in-PN and 50% RI-in-PN + 50% s.c. GI showed essentially no difference in MBG in patients receiving TPN and could be substituted for each other, which is consistent with previous meta-analyses and original studies (9, 22, 37, 39).

A *post hoc* analysis of the NICE-SUGAR study showed that patients with hypoglycemia had a higher risk of death than patients without hypoglycemia (46) and that the risk of hypoglycemia increased when patients received insulin infusion (37). Therefore, the incidence of hypoglycemia is a very important safety indicator when assessing different insulin infusion modalities in the treatment of TPN-associated hyperglycemia. Interestingly, the 6 interventions evaluated in this study were essentially equivalent in terms of hypoglycemia incidence. A prospective study enrolling 67 patients conducted by Hakeam et al. (9) in 2017 showed no difference in hypoglycemia incidence between s.c. GI and RI-in-PN in diabetic patients treated with PN. Li et al. (3) also showed that MSII and CSII had comparable hypoglycemia incidence in patients with prior diabetes receiving PN. The results of a prospective multicenter study conducted by Olveria et al. (22) in 2020 noted that nonsevere hypoglycemia was significantly higher with 50% RI-in-PN + 50% s.c. GI compared with RI-in-PN in noncritical patients with type 2 diabetes treated with TPN. However, due to the expansion of the sample size of the original study and the use of different test methods in the network meta-analysis, the results are more conservative, and the 95% CI range of the effect value is larger; consequently, negative results were ultimately obtained in our research. Previous studies have shown that the incidence of hypoglycemia was not significantly increased in CVII compared to MSII, despite the former taking longer to achieve glycemic targets (40).

In terms of hospital length of stay, the MSII group was the longest, which may be because most of the patients included in this study were perioperative patients. MSII yielded poor glycemic control and resulted in higher MBG levels, which led to an increased incidence of infectious complications, including SSI and delayed incisional healing (42), prolonging patients’ hospital stay. Patients in the CVII group had the shortest hospital stay. Although the control of MBG in CVII was not superior to that by RI-in-PN, s.c. GI, CSII and 50% RI-in-PN + 50% s.c. GI, its incidence of hyperglycemia (>180 mg/dL) was the lowest according to the results of this study. The research by Cheung et al. (14) showed that the incidence of infection in the 142–164 mg/dL blood glucose group was 2.8 times higher than that of the <124 mg/dL glucose group, and the incidence of infection in the >164 mg/dL group was 3.9 times higher than that of the <124 mg/dL group. In sum, hyperglycemia was closely associated with the incidence of any type of infection, and the higher the blood glucose was, the greater the risk of infection, and the correspondingly longer the hospital stay, which could explain the fact that the shortest hospital stay was in the CVII group.

Because of the limitations of the included studies, the incidence of hyperglycemia and incidence of SSI were compared for only 4 interventions, CVII, RI-in-PN, s.c. GI, and CSII. In terms of hyperglycemia. CVII was the best intervention with the lowest resultant incidence of hyperglycemia. According to Chinese guidelines for the prevention and treatment of type 2 diabetes in 2020 (53), CVII can be the preferred route for blood glucose control in critically ill patients, those undergoing emergency surgery and those undergoing large and medium-sized surgery because the insulin is rapidly absorbed, can achieve a higher plasma concentration after 1 h of administration, and can correct the hyperglycemic state more quickly (26), so the incidence of hyperglycemia in the CVII group is the lowest. Among the 4 interventions, the incidence of SSI was the lowest in the CSII group, but there was no significant difference with CVII. According to the previous description, the MBG was closely related to the incidence of SSI, and the higher the blood glucose, the greater was the risk of infection. CSII intervention was consistent with the characteristics of physiological insulin secretion, resulting in a lower MBG, while the incidence of hyperglycemia (>180 mg/dL) was higher than that of CVII; therefore, the incidence of SSI was essentially comparable and lower in both methods. In this study, the mean total daily insulin doses of 3 interventions, CVII, RI-in-PN and 50% RI-in-PN + 50% s.c. GI, were compared, with the CVII group having the lowest mean total daily insulin dose. It is well known that when patients have elevated blood glucose and need to increase insulin administration, CVII is rapidly absorbed, and with the same dosage of insulin, CVII can control hyperglycemia faster than other regimens, so the insulin dosage is relatively low.

This study has several strengths. First, to our knowledge, it is the first to quantitatively compare different insulin infusion methods in the treatment of PN-associated hyperglycemia, which can more accurately assess the safety and efficacy between different insulin infusion modalities and remedy the deficiencies in current published studies. Second, six commonly used insulin regimens were included in this study for analysis, which is the first comprehensive comparison of the insulin infusion methods currently used in clinical practice, thus helping clinicians to select the optimal insulin regimen individually when managing patients with different clinical characteristics. In addition, six clinical outcomes were included for analysis in this study, which is the first comprehensive, multifaceted assessment of the advantages and disadvantages of different insulin infusion routes. Furthermore, we conducted an extensive literature search and included more and newer literature than other studies. Finally, glycemic management in patients receiving PN therapy is very important and urgently needs to be addressed during clinical practice, but there are few relevant meta-analysis results, which shows the special significance of this study.

This study also has some limitations: (i) because of the small number of studies, 4 retrospective studies were not excluded from this study, which may have influenced the homogeneity, similarity and consistency required by the network meta-analysis; (ii) there are some differences in glycemic management between critically ill patients and patients in general wards; however, a subgroup analysis of critically ill patients could not be performed in this study due to the small number of critically ill patients included, and similarly, a subgroup analysis with/without diabetes was not possible; (iii) most of the included studies did not specify the formulation of TPN, which may be a potential confounding factor; (iv) the small sample size, especially for the 50% RI-in-PN + 50% s.c. GI, s.c. GI and MSII intervention measures and the short study duration of some of the studies may have led to false-negative results in the assessment of the impact on clinical outcomes; therefore, more large-sample studies are needed to verify the stability of the results. (v) The majority of the included studies were single-center studies, and the results may have some bias. (vi) This study included several non-SCI articles for analysis. Although the literature quality evaluation results indicated that they were high-quality studies, it cannot be denied that they may have caused some bias in the research results. Therefore, the conclusions of our research can provide a certain reference value for clinical practice, and they still need to be verified by RCTs with larger samples.

## 5. Conclusion

Overall, current literature shows that for the treatment of TPN-associated hyperglycemia, CVII is the most effective, reducing the incidence of hyperglycemia and shortening the length of hospital stay without increasing the incidence of hypoglycemia. MSII is the least effective, leading to higher blood glucose and longer hospital stays. RI-in-PN, CSII, s.c. GI and 50% RI-in-PN + 50% s. c. GI are better in terms of efficacy and safety and can be substituted for each other. In practice, clinicians can choose the insulin infusion route individually according to the specific conditions of the patients and the degree of difficulty of the nursing work. We also look forward to prospective, large-scale randomized controlled studies to determine the efficacy and safety between different insulin infusion methods and provide evidence to optimize the optimal treatment strategies for PN-associated hyperglycemia.

## Author contributions

LC and NZ designed research, screened literature, extracted data, analyzed data, and wrote the manuscript. PZ designed research, checked data, analyzed data, and revised the manuscript. DZ and YZ participated in designing research and revised the manuscript. All authors contributed to the article and approved the submitted version.

## Funding

This research received four fundings: Shaanxi Provincial People’s Hospital Science and Technology Development Incubation Funding (2022YJY-55), Pharmaceutical Services Research Fund of Shaanxi Health Care Association (KY-2023-01-YX-04), Traditional Chinese Medicine Inheritance and Innovation and Key Scientific Research Project for “Qin” Development (2021-02-zz-015), Natural Science Foundation of Shaanxi Province (2022JM-605).

## Conflict of interest

The authors declare that the research was conducted in the absence of any commercial or financial relationships that could be construed as a potential conflict of interest.

## Publisher’s note

All claims expressed in this article are solely those of the authors and do not necessarily represent those of their affiliated organizations, or those of the publisher, the editors and the reviewers. Any product that may be evaluated in this article, or claim that may be made by its manufacturer, is not guaranteed or endorsed by the publisher.

## References

[1] Klek S Krznaric Z Gundogdu RH Chourdakis M Galas A. Prevalence of malnutrition in various political, economic, and geographic settings. JPEN J Parenter Enteral Nutr. (2015) 39:200–10. doi: 10.1177/0148607113505860, PMID: 24190900

[2] MM MM Nystrom E Braunschweig C Miles J Compher C American Society for Parenteral and Enteral Nutrition (A.S.P.E.N.) Board of Directors; American Society for Parenteral and Enteral Nutrition. A.S.P.E.N. clinical guidelines: nutrition support of adult patients with hyperglycemia. JPEN J Parenter Enteral Nutr. (2013) 37:23–36. doi: 10.1177/0148607112452001 22753619

[3] Li FF Zhang WL Liu BL Zhang DF Chen W Yuan L. Management of glycemic variation in diabetic patients receiving parenteral nutrition by continuous subcutaneous insulin infusion (CSII) therapy. Sci Rep. (2018) 8:5888. doi: 10.1038/s41598-018-24275-5, PMID: 29651052 PMC5897521

[4] Truong S Park A Kamalay S Hung N Meyer JG Nguyen N. Glycemic control in adult surgical patients receiving regular insulin added to parenteral nutrition vs insulin glargine: a retrospective chart review. Nutr Clin Pract. (2019) 34:775–82. doi: 10.1002/ncp.10252, PMID: 30693980

[5] Herrmann GKE Richter CH Fehmann A. Glucagon-like peptide-1 and glucose-dependent insulin-releasing polypeptide plasma levels in response to nutrients. Digestion. (1995) 56:117–26. doi: 10.1159/000201231 7750665

[6] Verçoza Viana M Verçoza Viana L Tavares AL de Azevedo MJ. Insulin regimens to treat hyperglycemia in hospitalized patients on nutritional support: systematic review and meta-analyses. Ann Nutr Metab. (2017) 71:183–94. doi: 10.1159/000481355 29017173

[7] Boughton CK Bally L Martignoni F Hartnell S Hovorka R. Articles fully closed-loop insulin delivery in inpatients receiving nutritional support: a two-centre, open-label, randomised controlled trial. Lancet Diabetes Endocrinol. (2019) 7:368–77. doi: 10.1016/S2213-8587(19)30061-0, PMID: 30935872 PMC6467839

[8] Marathe CS Rayner CK Bound M Checklin H Standfield S Wishart J. Small intestinal glucose exposure determines the magnitude of the incretin effect in health and type 2 diabetes. Diabetes. (2014) 63:2668–75. doi: 10.2337/db13-1757, PMID: 24696447

[9] Hakeam HA Mulia HA Azzam A Amin T. Glargine insulin use versus continuous regular insulin in diabetic surgical noncritically ill patients receiving parenteral nutrition: randomized controlled study. JPEN J Parenter Enteral Nutr. (2017) 41:1110–8. doi: 10.1177/0148607116644710, PMID: 27091835

[10] Laesser CI Cumming P Reber E Stanga Z Muka T Bally L. Management of glucose control in noncritically ill, hospitalized patients receiving parenteral and/or enteral nutrition: a systematic review. J Clin Med. (2019) 8:935–52. doi: 10.3390/jcm8070935, PMID: 31261760 PMC6678336

[11] Olveira G Tapia MJ Ocón J Cabrejas-Gómez C Ballesteros-Pomar MD Vidal-Casariego A. Parenteral nutrition-associated hyperglycemia in non-critically ill inpatients increases the risk of in-hospital mortality (multicenter study). Diabetes Care. (2013) 36:1061–6. doi: 10.2337/dc12-1379, PMID: 23223407 PMC3631871

[12] Pasquel FJ Spiegelman R McCauley M Smiley D Umpierrez D Johnson R. Hyperglycemia during total parenteral nutrition: an important marker of poor outcome and mortality in hospitalized patients. Diabetes Care. (2010) 33:739–41. doi: 10.2337/dc09-1748, PMID: 20040658 PMC2845017

[13] Lin L-Y Lin H-C Lin H-D Lee P-C Ma W-Y. Hyperglycemia correlates with outcomes in patients receiving total parenteral nutrition. Am J Med Sci. (2007) 333:261–5. doi: 10.1097/MAJ.0b013e3180536b26, PMID: 17505165

[14] Cheung NW Napier B Zaccaria C Fletcher JP. Hyperglycemia is associated with adverse outcomes in patients receiving total parenteral nutrition. Diabetes Care. (2005) 28:2367–71. doi: 10.2337/diacare.28.10.2367, PMID: 16186264

[15] American Diabetes Association. 13. Diabetes care in the hospital. Diabetes Care. (2016) 39:S99–S104. doi: 10.2337/dc16-S016 26696689

[16] Yang L-L Bian X-J Yu F. Glucose control in non-critically ill hospitalized patients on parenteral nutrition therapy. Pharm Prog. (2016) 40:137–40.

[17] Gosmanov AR Umpierrez GE. Management of hyperglycemia during enteral and parenteral nutrition therapy. Curr Diab Rep. (2013) 13:155–62. doi: 10.1007/s11892-012-0335-y 23065369 PMC3746491

[18] McCulloch A Bansiya V Woodward JM. Addition of insulin to parenteral nutrition for control of hyperglycemia. JPEN J Parenter Enteral Nutr. (2018) 42:846–54. doi: 10.1177/0148607117722750, PMID: 28792863

[19] Jakoby MG Nannapaneni N. An insulin protocol for management of hyperglycemia in patients receiving parenteral nutrition is superior to ad hoc management. JPEN J Parenter Enteral Nutr. (2012) 36:183–8. doi: 10.1177/0148607111415628 21825091

[20] Hsu CW. Glycemic control in critically ill patients. World J Crit Care Med. (2012) 1:31–9. doi: 10.5492/wjccm.v1.i1.31, PMID: 24701399 PMC3956063

[21] Olveira G Tapia MJ Ocón J Cabrejas-Gómez C Ballesteros-Pomar MD Vidal-Casariego A. Prevalence of diabetes, prediabetes, and stress hyperglycemia: insulin therapy and metabolic control in patients on total parenteral nutrition (prospective multicenter study). Endocr Pract. (2015) 21:59–67. doi: 10.4158/EP13441.OR 25148810

[22] Olveira G Abuín J López R Herranz S García-Almeida JM García-Malpartida K. Regular insulin added to total parenteral nutrition vs subcutaneous glargine in non-critically ill diabetic inpatients, a multicenter randomized clinical trial: INSUPAR trial. Clin Nutr. (2020) 39:388–94. doi: 10.1016/j.clnu.2019.02.036, PMID: 30930133

[23] Umpierrez GE Hellman R Korytkowski MT Kosiborod M Maynard GA Montori VM. Management of hyperglycemia in hospitalized patients in non-critical care setting: an endocrine society clinical practice guideline. J Clin Endocrinol Metabol. (2012) 97:16–38. doi: 10.1210/jc.2011-2098, PMID: 22223765

[24] Jadad AR Moore RA Carroll D Jenkinson C Reynolds DJM Gavaghan DJ. Assessing the quality of reports of randomized clinical trials: is blinding necessary? Control Clin Trials. (1996) 17:1–12. doi: 10.1016/0197-2456(95)00134-4 8721797

[25] Stang A. Critical evaluation of the Newcastle–Ottawa scale for the assessment of the quality of nonrandomized studies in meta-analyses. Eur J Epidemiol. (2010) 25:603–5. doi: 10.1007/s10654-010-9491-z, PMID: 20652370

[26] Han L-O Han W-W Duan X-Q. Application of continuous subcutaneous insulin infusion to diabetic patients after abdominal major operation during the time of total parenteral nutrition. Chin J Emerg Med. (2006) 26:664–6. doi: 10.3969/j.issn.1002-1949.2006.09.009

[27] Chen J-S Luan X-J Hu L-D Zhong S-L Pan W-S. Application of insulin pump in total parenteral nutrition of diabetes patients. Pract Gen Med. (2007) 5:773–4. doi: 10.3969/j.issn.1674-4152.2007.09.012

[28] Huang D-H Lian G-Y Chen X Li W-L. Insulin pump is applied to the nursing of diabetes patients with total parenteral nutrition. Lingnan J Emerg Med. (2009) 14:408–9. doi: 10.3969/j.issn.1671-301X.2009.05.038

[29] Long H Lin Z-C Wang Y-N Lu H-P Si T-D-R. Randomized controlled study on the effect of insulin infusion mode on its activity, concentration and blood glucose in parenteral nutrition. Chin J Surg. (2009) 47:286–8. doi: 10.3760/cma.j.issn.0529-5815.2009.04.014 19570393

[30] Lan G-H Xie Y-Z Fang M Lin Z-W. Blood glucose control of postoperative parenteral nutrition in patients with gastrointestinal cancer in diabetes. Mod Hosp. (2010) 10:33–4. doi: 10.3969/j.issn.1671-332X.2010.03.014

[31] Wang Y. Observation on the effect of insulin administration on blood glucose control in postoperative total parenteral nutrition in patients with gastric cancer and diabetes. Tianjin Nurs. (2010) 18:315–6. doi: 10.3969/j.issn.1006-9143.2010.06.003

[32] Gao S Liu C-Y. Comparison of different blood glucose control methods in parenteral nutrition for patients with gastric cancer and diabetes. Gen Nurs. (2011) 9:956–7. doi: 10.3969/j.issn.1674-4748.2011.011.009

[33] Zheng L Liao Y-G Zheng H-Y Li Y-L Cheng C-M. Clinical comparison of insulin pump and insulin glargine in total parenteral nutrition for diabetes patients after operation. Chin Foreign Health Abstr. (2011) 8:86–7. doi: 10.3969/j.issn.1672-5085.2011.13.073

[34] Liu J Ma Y-J Gao S-G Jiang H-W. Comparison of different blood glucose control methods in patients with gastric cancer and diabetes. Chin J Pract Med. (2012) 39:98–9. doi: 10.3760/cma.j.issn.1674-4756.2012.18.047

[35] Duan Y-W Zhao Y. Application of insulin saline micropump during parenteral nutrition after abdominal surgery in patients with diabetes. Chin Med Sci. (2013) 3:24–35.

[36] Shi E-H. Comparison of different blood glucose control methods in parenteral nutrition for patients with gastric cancer and diabetes. N World Diab. (2015) 10:92–3. doi: 10.16658/j.cnki.1672-4062.2015.10.119

[37] Oghazian MB Javadi MR Radfar M Torkamandi H Sadeghi M Hayatshahi A. Effectiveness of regular versus glargine insulin in stable critical care patients receiving parenteral nutrition: a randomized controlled trial. Pharmacotherapy. (2015) 35:148–57. doi: 10.1002/phar.1546, PMID: 25689245

[38] He Z Wu Y. Comparative study on the effect of two methods to control postoperative hyperglycemia of pancreatic cancer. Lab Med Clin. (2016) 13:2931–2. doi: 10.3969/j.issn.1672-9455.2016.20.033

[39] Wang W Ni W-J Zhao X-M. Clinical efficacy of different insulin administration modes during parenteral nutrition in diabetes. Chinese. J Emerg Med. (2018) 38:77–8. doi: 10.3969/j.issn.1002-1949.2018.z1.067

[40] Neff K Donegan D MacMahon J O’Hanlon C Keane N Agha A. Management of parenteral nutrition associated hyperglycaemia: a comparison of subcutaneous and intravenous insulin regimen. Ir Med J. (2014) 107:141–3. PMID: 24908857

[41] Yang L-L Bian X-J Ge W-H Yu F. Comparison of clinical effects of different blood glucose regulation methods in patients with diabetes who received parenteral nutrition after abdominal surgery. J Clin Ration Drug use. (2016) 9:7–8. doi: 10.15887/j.cnki.13-1389/r.2016.05.004

[42] Wu S Fan Y-Y Qiu Y-D Xie M Fu Q-M. The application of two blood glucose regulation methods in parenteral nutrition support after pancreatoduodenectomy. Gen Nurs. (2022) 20:790–2. doi: 10.12104/j.issn.1674-4748.2022.06.019

[43] Boughton CK Bally L Martignoni F Hartnell S Herzig D Vogt A. Fully closed-loop insulin delivery in inpatients receiving nutritional support: a two-centre, open-label, randomised controlled trial. Lancet Diabetes Endocrinol. (2019) 7:368–77. doi: 10.1016/S2213-8587(19)30061-0, PMID: 30935872 PMC6467839

[44] Olveira G Tapia MJ Ocon J Cabrejas-Gomez C Ballesteros-Pomar MD Vidal-Casariego A. Hypoglycemia in noncritically ill patients receiving total parenteral nutrition: a multicenter study. (study group on the problem of hyperglycemia in parenteral nutrition; nutrition area of the Spanish Society of Endocrinology and Nutrition). Nutrition. (2015) 31:58–63. doi: 10.1016/j.nut.2014.04.023, PMID: 25441588

[45] Carey M Boucai L Zonszein J. Impact of hypoglycemia in hospitalized patients. Curr Diab Rep. (2013) 13:107–13. doi: 10.1007/s11892-012-0336-x 23065370

[46] Investigators N-SS. Hypoglycemia and risk of death in critically ill patients. N Engl J Med. (2012) 367:1108–18. doi: 10.1056/NEJMoa1204942 22992074

[47] Forchielli ML Bongiovanni F Platé L Piazza G Puggioli C D’Alise A. Insulin instability in parenteral nutrition admixtures. JPEN J Parenter Enteral Nutr. (2018) 42:907–12. doi: 10.1002/jpen.1024, PMID: 30001464

[48] Mühlebach S. Practical aspects of multichamber bags for total parenteral nutrition. Curr Opin Clin Nutr Metab Care. (2005) 8:291–5. doi: 10.1097/01.mco.0000165008.47073.ba, PMID: 15809532

[49] Mühlebach S Franken C Stanga Z. Working group for developing the guidelines for parenteral nutrition of The German Association for Nutritional Medicine. Practical handling of AIO admixtures—guidelines on parenteral nutrition, chapter 10. Ger Med Sci. (2009):7. doi: 10.3205/000077 PMC2795373 20049073

[50] Rong Y-H Gu W Geng J-L Zhang X-K Zhang R. Comparison of blood glucose variability in T2DM patients with different subcutaneous insulin injection schemes. Chin J Mod Med. (2017) 27:5. doi: 10.3969/j.issn.1005-8982.2017.17.022

[51] Bai C-H Chen L Fu Q-M. Meta-analysis of the effect of different insulin infusion methods in parenteral nutrition. PLA. Nurs J. (2018) 35:27–32.

[52] Chinese Diabetes Society. Guideline for the prevention and treatment of type 2 diabetes mellitus in China (2020 edition). Chin J Diab Mellit. (2021) 13, 315–409. doi: 10.3760/cma.j.cn115791-20210221-00095

[53] Drincic AT Knezevich JT Akkireddy P. Nutrition and hyperglycemia management in the inpatient setting (meals on demand, parenteral, or enteral nutrition). Curr Diab Rep. (2017) 17:1–12. doi: 10.1007/s11892-017-0882-3 28664252

[54] Lenhard MJ Reeves GD. Continuous subcutaneous insulin infusion: a comprehensive review of insulin pump therapy. Arch Intern Med. (2001) 161:2293–300. doi: 10.1001/archinte.161.19.2293 11606144

